# Development and Implementation of a Three-dimensional Printed Knee Joint Simulation Model Using the Consolidated Framework for Implementation Research: Addressing Local Simulation Needs

**DOI:** 10.7759/cureus.4364

**Published:** 2019-04-02

**Authors:** Eugene Krustev, Adam Dubrowski

**Affiliations:** 1 Medical Education and Simulation, Memorial University of Newfoundland, St. John's, CAN; 2 Emergency Medicine, Memorial University of Newfoundland, St. John's, CAN

**Keywords:** 3d printing, simulation

## Abstract

Background

Knee joint injections and aspirations are essential procedures for medical students, residents, and primary care physicians to master. Simulation-based training has been shown to improve learner confidence and performance scores in knee joint injections. Current knee joint simulators are expensive, ranging from hundreds to thousands of dollars. Using three-dimensional (3D) printing and gel layering technology, we designed and manufactured an inexpensive simulator. The aim of this implementation study was to gather the opinions of local simulation specialists and administrators regarding the simulator's curricular implementation.

Methods

Using the Consolidated Framework for Implementation Research (CFIR), we developed a 31-item implementation survey. It was administered to local simulation specialists and administrators. The purpose of the survey was to identify the aspects of the simulator that they deemed important to the implementation process, as well as obtain their qualitative feedback about the design.

Results

In total, three participants completed the survey. There were 16 survey items that were rated as very important, including local manufacturing, appropriate planning, internal development, evidence-based development, and reasonable costs. Another nine items were deemed important, including the adaptability of the product and ability to test the product. The simulation specialists also expressed some concerns they had with the design of the simulator and made suggestions about how we could address these concerns.

Conclusions

Local development and manufacturing, coupled with appropriate pre-implementation planning and efficacy evidence, were selected as factors that would potentially contribute to the success of the implementation of the simulator in the local curriculum.

## Introduction

A recent survey showed that 86% of participating Canadian family physicians agree that knee joint injection and aspiration skills, used to aid in the diagnosis and treatment of knee joint diseases, should be a core competency and 91% of those physicians performed these procedures in their practice [1]. Conversely, in a study from the United Kingdom, only 65% of primary care physicians surveyed offered intra-articular steroid injections. Of those who did not offer the service, 64% reported insufficient training as their reason [2]. When a group of American internal medicine and family medicine residents was surveyed, they rated their comfort with performing joint injections as 4.4 ± 2.5 out of 10 on a visual analog scale [3]. These findings support the fact that family physicians should be capable of performing knee joint injections and that many primary care physicians in Canada offer this service; however, further training is needed at the undergraduate and postgraduate levels in order to increase confidence and proficiency among physicians. One method that could be used to teach medical trainees how to properly perform knee joint injections and aspirations is through the use of simulation models.

In the modern medical school curriculum, simulation has become an important part of training future physicians. Invasive techniques like injections, suturing, and surgical skills are safely taught using simulation models. For example, in one study, residents and fourth-year medical students were divided into three groups. The control group received no training in joint injections, the second group of participants only received a classroom-based teaching session, and a third group received the classroom session and a -simulation-based practical session. As expected, both groups who received the teaching sessions performed better than the control group on the knowledge-based written test. Furthermore, the group who received both the classroom and the simulation sessions performed better on the practical test [4]. In another study, physician comfort scores significantly improved after undergoing joint injection simulation training. This effect was greatest in first-year residents, which highlights the fact that inexperienced learners can benefit greatly from simulation-based injection workshops [3].

Unfortunately, simulation models are expensive, ranging from hundreds to thousands of dollars, which can significantly limit the access that medical trainees have to these tools. Our aim was to develop a locally manufactured and affordable simulation model and, therefore, increase the accessibility of simulation-based skills training curricula. Using 3D printing and gel layering technologies, we designed a prototype model for learning knee joint injections and aspirations. After developing the prototype, the next step was to begin the pre-implementation process and gain feedback from local simulation administrators and specialists.

To accomplish this simulation initiative, we used a modified version of Damschroder's *Consolidated Framework For Implementation Research (CFIR) *[5]. In the original conception, CFIR is composed of five main categories: 1) intervention characteristics, 2) outer setting, 3) inner setting, 4) characteristics of individuals, and 5) process. These main constructs are then subdivided into multiple constructs. For the purposes of this work, we selected 31 CFIR constructs that were most applicable to the implementation of our knee joint model. Then, we adapted these constructs to better fit this project and developed a survey to assess the importance of each of these constructs.

Therefore, the aim of this project was to use a CFIR-based survey to gather the opinions of our local Clinical Learning and Simulation Centre (CLSC) staff and leaders about the potential for the implementation of this alternative knee-joint model into undergraduate and postgraduate medical-based curricula, as well as to improve the locally developed and manufactured knee joint model.

## Materials and methods

**Figure 1:**
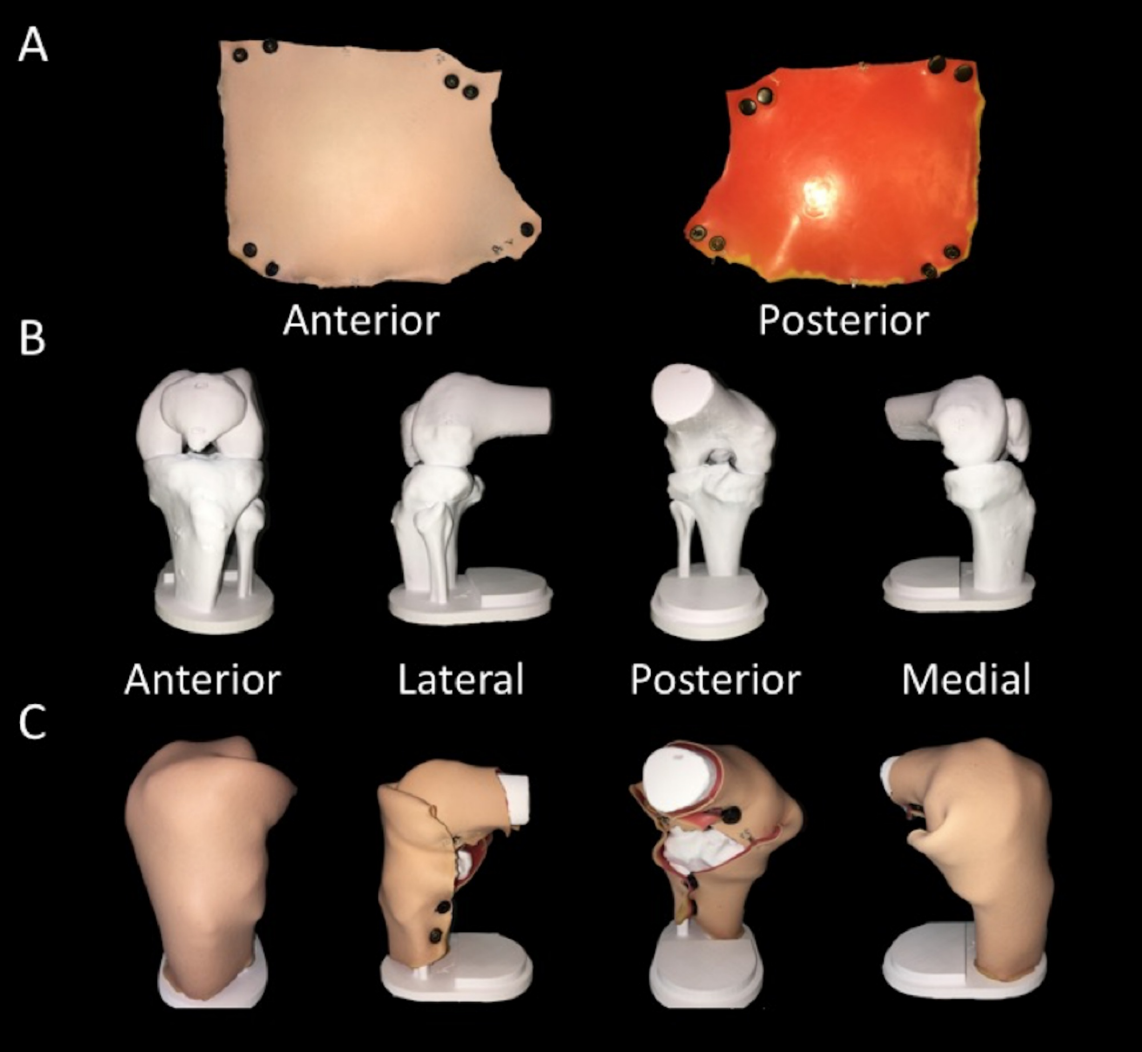
First prototype of knee joint model. A) Anterior and posterior views of outer skin. B) Bony frame of knee joint model. C) Assembled knee joint model.

This study was exempt from full ethics review and classified as program/product development and evaluation (Health Research Ethics Authority reference number: 20192928). Participants were the simulation specialists and administrators of the local simulation center: the Clinical Learning and Simulation Centre (CLSC). Five participants were approached, and three completed the survey. The participants were provided with the knee joint model prototype (Figure 1), as well as the modified CFIR (referred to as the implementation survey) (Table 1). Each of the 31 implementation survey items was ranked on a Likert scale from 1 to 5 (1 - very unimportant, 2 - unimportant, 3 - somewhat important, 4 - important, and 5 - very important). The participants were asked to explore the features of the prototype model and use the survey to rank each CFIR construct/item as they pertain to the model. The participants were also encouraged to make comments on the survey about the simulator. At the end of the survey, the staff were asked to provide any additional comments that they wanted to express about the model.

**Table 1:** Implementation survey that was completed by the participants

Please rate how important you think each of these factors would be relative to your adoption of a product, as well as the success of that product. 1 – very unimportant, 2 – unimportant, 3 – somewhat important, 4 – important, 5 – very important.					
MODEL CHARACTERISTICS					
How important is it that the simulation model is developed internally?	1	2	3	4	5
How important is it that the product is manufactured internally, and that replacement parts can be acquired and manufactured locally?	1	2	3	4	5
How important is it that the simulation model is supported by research derived evidence?	1	2	3	4	5
How important is it that the simulation model provides a technical advantage over similar models (i.e. has more functions)?	1	2	3	4	5
How important is it that this model is more affordable than other similar models?	1	2	3	4	5
How important are the implementation costs of the simulation model?	1	2	3	4	5
How important is it that the simulation model can be adaptable, so that it can be tailored or reinvented to fit learner needs?	1	2	3	4	5
How important is it that the simulation model can be tested on a small scale, so you can adapt its function if needed?	1	2	3	4	5
How important is the simulation model’s design, packaging, and presentation?	1	2	3	4	5
LEARNER CHARACTERISTICS					
How important is it that the simulation model addresses the needs of learners at all levels of training (i.e. medical students, residents, staff)?* *If you answered ≤ 2, which learners should the model be tailored for?	1	2	3	4	5
How important is it that the model assesses the needs of learners from other health care professions (e.g. nurses, pharmacists, physiotherapists, occupational therapists, etc.)?	1	2	3	4	5
How important is it that learners at your own organization are aware of the model?	1	2	3	4	5
How important is it that learners at other organizations are aware of this model?	1	2	3	4	5
How important is it that there are external policies and incentives to support the adoption of the simulation model by learners (e.g., incorporation of the model into the curriculum, availability of the model in an independent learning room, allotted time in learner’s schedule for simulation learning)?	1	2	3	4	5
STAFF CHARACTERISTICS					
How important are staff/faculty attitudes toward evidence-based models?	1	2	3	4	5
How important are staff/faculty beliefs in their own capabilities to do what is needed to implement the new model?	1	2	3	4	5
How important are staff/faculty familiarity with the use of simulation models for medical teaching?	1	2	3	4	5
INNER SETTING					
How important are member views towards the use of simulation products in regards to the education of healthcare professionals?	1	2	3	4	5
How important is the degree of fit between this product and other products in use at the simulation center?	1	2	3	4	5
How important is it that within your organization there is a shared perception of the priority of implementing this product as opposed to other similar products?	1	2	3	4	5
In order for this product to succeed, how important is it that your organization offers incentives for promoting internally developed simulation products?	1	2	3	4	5
For this product to succeed, how important is the degree to which the goals of using internally developed products are clearly communicated, acted upon, and fed back to staff within your organization?	1	2	3	4	5
In order for this product to succeed, how important is it that within your organization there is commitment and involvement of leaders to the implementation of the internally developed products?	1	2	3	4	5
How important is the level of resources dedicated for implementation and on-going operations related to this product including money, training, education, physical space, and time?	1	2	3	4	5
How important is it that you can easily access information and knowledge about this product?	1	2	3	4	5
PROCESS					
How important is the degree of planning prior to implementation of this product?	1	2	3	4	5
In order for this product to succeed, how important is it to attract/interest key individuals through strategies such communication, education, and training?	1	2	3	4	5
For the success of this simulation product, how important is it to involve people in your organization who have formal or informal influence on the attitudes and beliefs of their colleagues with respect to implementing the tool?	1	2	3	4	5
How important is it to involve people from outside your organization who can formally influence or facilitate the implementation of this new product?	1	2	3	4	5
How important is it that the implementation of the product is carried out or accomplished according to a specific plan?	1	2	3	4	5
How important is it that implementation is informed by quantitative and qualitative feedback about the progress and quality of implementation, as well as regular personal and team debriefing about progress and experience?	1	2	3	4	5
Thank you for taking the time to complete this questionnaire! If you have any other suggestions or would like to elaborate on any of your answers for any of the questions above, please use the following few lines to do so:					

All survey data were compiled and organized by frequencies of ranking. Furthermore, the qualitative data from the questionnaire were also compiled.

## Results

In total, there were 16 construct items that were rated as very important. Based on these results, it is very important that the model is manufactured internally and that replacement parts can be acquired and manufactured locally (Question 2 of the survey) and that there is an appropriate degree of planning prior to the implementation of this model (Question 26). Furthermore, the model should be developed internally (Question 1), supported by evidence (Question 3), more affordable than other similar models (Question 5), and have reasonable implementation costs (Question 6). The CLSC staff also identified that in order for this model to succeed, it is very important that the medical staff have positive attitudes towards evidence-based models (Question 15) and are familiar with the use of simulation models for medical teaching (Question 17). Informing physician instructors of the importance of using internally developed tools (Question 22), as well as having organizational leaders that are committed to promoting the use of internally developed tools (Question 23), were also rated as very important. Additionally, adequate resources (Question 24), easily accessible information (Question 25), attracting key individuals (Question 27), involving influential members of our organization (Question 28), and having a plan for the implementation were all rated as very important (Question 30). Lastly, the CLSC staff also agreed that it would be very important for the implementation of this model to be informed by quantitative and qualitative feedback, as well as individual and team debriefings (Question 31).

Based on the results of our survey, there were nine items that were rated as important. These important constructs were that the model should be adaptable to learner’s needs (Question 7), testable on a small scale (Question 8), and able to meet the needs of learners at all levels (Question 10). Furthermore, the staff at the CLSC also thought that the design, packaging, and presentation of the model were important (Question 9). Having policies and incentives to support the adoption of this simulation model by learners (Question 14) and that staff are confident in their ability to implement this model (Question 16) were also rated important. Lastly, it was rated as important that members within our organization have positive views toward a simulation model (Question 18), that this model fits well with other models in the CLSC (Question 19), and that there is a shared perception within the organization to implement this model rather than a similar model (Question 20).

In addition to the quantitative results of the survey, the CLSC staff were given the opportunity to provide qualitative, open-ended, written feedback about the model and several of these comments were made about particular survey questions. The CLSC staff highlighted that internal development (i.e. the model developed in consultation with local experts) and appropriate planning before implementation were the most important constructs listed above. When asked if this model should be applicable to learners at all levels (Question 10), the CLSC staff rated this as important but also stated that the model should primarily be tailored to “residents (and) practicing doctors.” Furthermore, when asked if they thought it is important that learners within the faculty of medicine are aware of this model (Question 12), the CLSC staff did not answer this question but rather commented that when this model is developed and tested, this would be important.

In an open comments section, the participants also had several comments concerning the model’s structural features. Firstly, they suggested that the model should not be on a 90-degree angle and that a slightly flexed position would be more appropriate. Furthermore, they also commented that the stand on which the model is mounted will require some modifications to keep it fixed in place when in use. They also mentioned that the skin should be a sleeve and that the snap buttons would not work adequately. Lastly, the CLSC staff also stated that a vestibule is needed to store the simulated synovial fluid during injection and aspiration.

**Figure 2:**
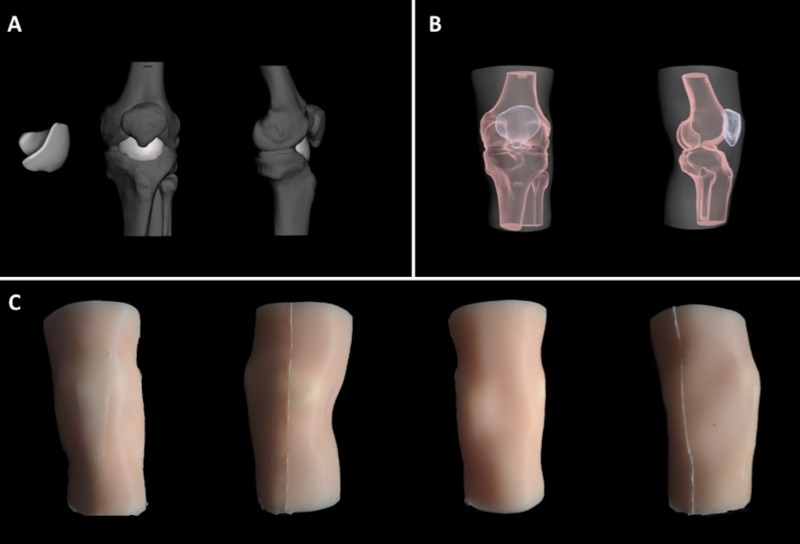
Steps in the design of the second prototype of knee joint model that include changes based on input from CLSC staff. A) Step 1: Computer model of new internal synovial fluid vestibule. B) Step 2: Computer model of bony frame within new molded gel sleeve. C) Step 3: Full surface view of the model. Panel A shows the computer model of the new internal synovial fluid vestibule, as well as its positioning within the new bony frame. From left to right: Image 1 shows the isolated synovial vestibule, image 2 shows the vestibule embedded in the joint in the coronal plane, and image 3 shows the vestibule embedded in the joint in the sagittal plane. Panel B depicts the computer model of the bony frame, including the patella in both coronal (image 1) and sagittal (image 2) planes within the new molded gel sleeve. Finally, panel C depicts the surface view of the assembled, physical model with the outer gel sleeve. Images 1 to 4 (from left to right) demonstrate the coronal-anterior, sagittal-medial, coronal-posterior, and sagittal-lateral views of the model. CLSC: Clinical Learning and Simulation Centre

Based on the feedback provided, the model was revised (Figure 2). The current revised model includes a bony frame that is fixed at a 15-degree angle. Although these two iterations of the model were rigid (i.e. they could only accommodate 15 or 90-degree flexion), our next improvements will focus on a fully articulating knee that can accommodate a range of flexion angles. Furthermore, we have designed a patellar ligament with three clips, which allows the secure attachment to the femur (superior), patella (middle) and tibia (distal), as well as a small degree of horizontal movement. A silicone sleeve has been designed using a 3D printed mold to simulate the surrounding musculature and skin. Finally, the synovial vestibule as a fluid container and has been made out of self-sealing silicone material. This will allow the replenishment of fluids for multiples attempts. Expert- and user-based face and construct validation study of this new model is currently underway.

## Discussion

The use of simulation is an important part of learning clinical and procedural skills in healthcare [6]. During the first two years of training, medical students practice history taking and physical exams on standardized patients and simulation mannequins. However, the costs of simulation can be high [7]. The use of innovative manufacturing techniques locally, such as 3D printing, can provide parsimonious solutions [8]. The aim of this project was to develop an affordable and easily reproducible knee joint simulation model using 3D printing techniques and to conduct an assessment before the simulator is implemented into the medical school curriculum to help teach students how to properly perform knee joint injections and aspirations.

Based on the results of our survey, internal development and manufacturing are very important. To date, this simulator has been entirely designed, manufactured, and tested locally. As development continues, it is important that we continue to rely on locally available resources. Furthermore, as locally available manufacturing techniques continue to evolve, the capabilities of this simulator should as well. Because evidence about the efficacy of this knee-joint simulator was rated high in the implementation survey, as the prototype continues to evolve, we will aim to test it on a larger scale, which will likely require recruiting participants from other medical schools. At the minimum, we will aim to address the face, content, and construct validity of this model in the immediate future [9].

Attitudes toward the use of simulation in medical education, as well as the specific use of this product within our organization, were also deemed important by the CLSC staff. In two separate studies, when vascular surgery and dermatology residents were surveyed about their attitudes towards the use of simulation in their training, 86%-93% of those surveyed reported it as beneficial [10-11]. Staff confidence in their ability to implement this model was also rated as important by the CLSC staff. This is similar to broader findings; when residency program directors and representatives were surveyed from each Canadian emergency medicine residency program in Canada, lack of dedicated faculty time and experience with simulation were listed as the main barriers to the use of simulation in their curriculum [12]. It would be interesting to see if similar barriers exist within our local facility and if increasing dedicated time and training for the use of simulation would help mitigate these barriers. Furthermore, as we continue to develop this knee joint model, it is important that we involve the appropriate medical staff in this process.

There were several suggestions about the design of the model. The first suggestion was that the model should be in a slightly flexed position, rather than in a 90-degree flexed position. Their reasoning for this suggestion is so that students can learn the medial and lateral approaches for knee injections and aspirations, as these techniques reduce the risk of iatrogenic meniscal injury. Using 3D printing software, we have rearranged the bones to be at 10-20 degree angle configurations for the bony frame in our new prototype (Figure 2). It was also noted that the skin and snap buttons will have a limited lifespan. We have developed a 3D printed gel mold and have developed a technique for casting a silicone gel sleeve of a leg around the bony frame; therefore, eliminating our need for button attachments (Figure 2). The CLSC staff also commented on the stand and suggested that it will need to keep it fixed in place; therefore, the new prototype now sits in the correct position. Furthermore, we have also developed a fluid compartment that can hold simulated synovial fluid and is self-sealing, which satisfies the CLSC staff’s request for an internal fluid-filled vestibule (Figure 2). However, the number of consecutive procedures before the leakage starts will be assessed in the next iteration of the model along with the face and content validities.

Model affordability and low implementation costs were rated as very important by the CLSC staff. Several commercial knee joint models offer more advanced feedback modalities, but at a significantly increased cost. Although the exact price of our final model has yet to be determined, our newest prototype would cost approximately $100 CAD to produce. It was noted in one study that the skin on the Sawbone injection models “tend(s) to deteriorate at the injection sites with repeated use” [3], and replacement skin for this model costs $140 USD. For our model, we could replace the entire gel sleeve for approximately $100 CAD and simply repair it with a layer of gel epoxide for a fraction of that price.

In summary, as suggested by Dubrowski and Dubrowski [13], we have followed a previously developed implementation model to construct an implementation survey with the hope to identify factors that will enable us to iteratively develop a simulator to be seamlessly implemented into undergraduate and post-graduate local curricula. In addition to several critical changes to the simulator, the survey provided us with a number of factors that we need to pay attention to in the future such as the generation of evidence to support the efficacy of the model, keeping costs down, and engaging local experts in the refinement of the simulator.

## Conclusions

The results of this study show that it is possible to design and develop a knee joint injection model using the manufacturing tools available within the Faculty of Medicine at the Memorial University of Newfoundland. By surveying the CLSC staff, several modifications to the model were suggested. By using these suggestions, we hope to continue developing a more realistic model with increased capabilities. Furthermore, using the CFIR, several constructs were identified as important to the development of this product. Further research is needed to learn what constructs the future users of this model (both clinician instructors and student learners) would like us to focus on.
